# Global Invader Impact Network (GIIN): toward standardized evaluation of the ecological impacts of invasive plants

**DOI:** 10.1002/ece3.1551

**Published:** 2015-06-30

**Authors:** Jacob N Barney, Daniel R Tekiela, Maria Noelia Barrios-Garcia, Romina D Dimarco, Ruth A Hufbauer, Peter Leipzig-Scott, Martin A Nuñez, Aníbal Pauchard, Petr Pyšek, Michaela Vítková, Bruce D Maxwell

**Affiliations:** 1 Department of Plant Pathology, Physiology, and Weed Science, Virginia TechBlacksburg, Virginia, 24061, USA; 2 CONICET, CENAC-APNFagnano 244, Bariloche, Argentina; 3 Grupo de Ecología de Poblaciones de Insectos (GEPI), INTA-CONICETModesta Victoria 4450, Bariloche, Argentina; 4 Department of Bioagricultural Sciences and Pest Management and Graduate Degree Program in Ecology, Colorado State University1177 Campus Delivery, Fort Collins, Colorado, 80523, USA; 5 Laboratorio de Ecotono, INIBIOMA, CONICET, Universidad Nacional del ComahueQuintral 1250, Bariloche, Argentina; 6 Laboratorio de Invasiones Biológicas (LIB), Facultad de Ciencias Forestales, Universidad de ConcepciónCasilla 160-C, Concepción, Chile; 7 Institute of Ecology and Biodiversity (IEB)Santiago, Chile; 8 Department of Invasion Ecology, Institute of Botany, The Czech Academy of SciencesCZ-252 43, Průhonice, Czech Republic; 9 Department of Ecology, Faculty of Science, Charles University in PragueViničná 7, CZ-128 44, Prague, Czech Republic; 10 Department of Land Resources and Environmental Sciences, Montana State UniversityBozeman, Montana, 59717, USA

**Keywords:** Coordinated distributed experiment, impact assessment, invasive plants, meta-analysis, natural experiment, research network, research protocol

## Abstract

Terrestrial invasive plants are a global problem and are becoming ubiquitous components of most ecosystems. They are implicated in altering disturbance regimes, reducing biodiversity, and changing ecosystem function, sometimes in profound and irreversible ways. However, the ecological impacts of most invasive plants have not been studied experimentally, and most research to date focuses on few types of impacts, which can vary greatly among studies. Thus, our knowledge of existing ecological impacts ascribed to invasive plants is surprisingly limited in both breadth and depth. Our aim was to propose a standard methodology for quantifying baseline ecological impact that, in theory, is scalable to any terrestrial plant invader (e.g., annual grasses to trees) and any invaded system (e.g., grassland to forest). The Global Invader Impact Network (GIIN) is a coordinated distributed experiment composed of an observational and manipulative methodology. The protocol consists of a series of plots located in (1) an invaded area; (2) an adjacent removal treatment within the invaded area; and (3) a spatially separate uninvaded area thought to be similar to pre-invasion conditions of the invaded area. A standardized and inexpensive suite of community, soil, and ecosystem metrics are collected allowing broad comparisons among measurements, populations, and species. The method allows for one-time comparisons and for long-term monitoring enabling one to derive information about change due to invasion over time. Invader removal plots will also allow for quantification of legacy effects and their return rates, which will be monitored for several years. GIIN uses a nested hierarchical scale approach encompassing multiple sites, regions, and continents. Currently, GIIN has network members in six countries, with new members encouraged. To date, study species include representatives of annual and perennial grasses; annual and perennial forbs; shrubs; and trees. The goal of the GIIN framework is to create a standard yet flexible platform for understanding the ecological impacts of invasive plants, allowing both individual and synthetic analyses across a range of taxa and ecosystems. If broadly adopted, this standard approach will offer unique insight into the ecological impacts of invasive plants at local, regional, and global scales.

## Introduction

Invasive plants are found in nearly every ecosystem on earth and are known to pose major threats to biodiversity, global and local economies, and ecosystem function (Mack et al. 2000; Wardle et al. 2011). Both their ubiquity and the potential breadth and magnitude of their direct and indirect impacts on life-sustaining ecosystem services make understanding the ecological impacts of invasive plants of broad societal relevance (Charles and Dukes 2007; Pejchar and Mooney 2009). Here, we define impact as a measurable change to an ecosystem property attributable to an individual species (Ricciardi et al. 2013; Jeschke et al. 2014). A large body of research exists characterizing the ecological impacts of invasive plants to a multitude of ecosystem pools and fluxes across many biomes and species (Vilà et al. 2011; Pyšek et al. 2012). The classification, magnitude, extent, directionality, and scale of impacts vary tremendously among species and ecosystems (Skurski et al. 2013, 2014). However, the taxonomic breadth of studies on invasive plant impacts is surprisingly limited, with only nine species accounting for 30% of all studies (Hulme et al. 2013). Additionally, the depth of our knowledge on impacts is similarly poor, with only about three different types of impacts examined in most studies (Hulme et al. 2013). Hundreds of studies have been aggregated to draw some broad conclusions on the impacts of invasive plants (Vilà et al. 2011; Pyšek et al. 2012), but the variation is often large, and the selection of metrics unclear and variable (Hulme et al. 2013). Additionally, meta-analyses of existing ecological data in general suffer from several limitations, including differences in method, scale, grain size, and impact metric quantification (Koricheva and Gurevitch 2014). While we collectively recognize that the impacts from invasive plants are important, and sometimes obvious, our evidence-based understanding is distressingly limited. Invasion science has an imperative to develop an understanding of the impacts of invasive plants based on sound empirical data, particularly now as budgets, perception, prioritization, and policy for their control hinge on this information (Simberloff et al. 2013).

Meeting this imperative requires standard and efficient methods that can be applied to diverse species and ecosystems, allowing meaningful comparisons, affording management prioritization, and facilitating policy setting to mitigate current and prevent future impacts. Coordinated distributed experiments (CDE) using standardized protocols applied globally offer the highest probability of advancing general ecological principles (Fraser et al. 2012; Sagarin and Pauchard 2012). Coordinated distributed experiments have been used with tremendous success with diverse focus including effects of nutrients, herbivores, soil moisture, CO_2_, and pollution on various ecosystem processes (Fraser et al. 2012). One of the best known and most productive distributed experiments is the Nutrient Network (also known as NutNet), which maintains >40 grassland sites globally. The power of this network has resulted in unprecedented insight into drivers of diversity–productivity relationships (e.g., Borer et al. 2014b), standing biomass–litter relationships (O’Halloran et al. 2013), and understanding exotic species dominance (Seabloom et al. 2013). There are at least two examples of coordinated research groups focused on plant invasions: the Global Garlic Mustard Field Survey (Colautti et al. 2014) and the Mountain Invasion Research Network (Pauchard et al. 2009). However, no coordinated research network is focused on the ecological impacts of invasive plants. We believe that using a network of globally distributed standardized experiments is the most effective approach to studying invasive plant impacts. Single studies and subsequent meta-analyses will always suffer from site-level effects, reducing robustness and the ability to generalize.

It has been widely demonstrated in a multitude of single studies that invasive plants can modify, among other things, native and exotic richness, soil nutrient pools and fluxes, microenvironments, disturbance regimes, and successional trajectories. However, as discussed above, rarely are many of these evaluated in a single system. Additionally, the methods used to identify these changes generally involve pairwise comparisons among the invasion and an uninvaded area or locations following invader removal (Kumschick et al. 2015). Both methods have advantages and disadvantages that have been discussed elsewhere (see Kumschick et al. 2015). The GIIN protocol is designed to overcome single study systems in the following ways that make it unique and the best available method to address invasive plant impacts: (1) recording many of the most commonly cited ecosystem pools and fluxes affected by invasive plants that are also important to ecosystem function; (2) using invaded, uninvaded, and removal plots allowing a variety of comparisons; (3) recording impact over time; (4) evaluating invader cover–impact relationships; (5) ability to incorporate environmental co-variation; (6) flexibility to incorporate socioeconomic variables; (7) identification of common or unique impacts among species, life forms, and habitats; (8) development of hypothesis testing for the mechanisms behind the impacts.

Here we present an experimental framework to serve as the foundation for a standard methodology to identify the ecological impacts of invasive plants and how those impacts scale spatially and temporally. The methods described are currently being used by the Global Invader Impact Network (GIIN), a developing coordinated distributed experiment centered on invasive plant impacts.

## Materials and Methods

### Conceptual considerations

The conceptual framework for GIIN includes the following premises:

1. By measuring the change in key species, community, and ecosystem parameters, impacts can be estimated (Vilà et al. 2011). The GIIN method relies in part on the assumption that it is possible to identify sites that have similar pre-invaded plant community and other environmental conditions adjacent to areas that are now invaded (Hejda et al. 2009).
2. Monitoring the dynamic of potential legacy effects from invasion in the plant community can give us insights into how to better manage and restore invaded ecosystems and will quantify the duration and extent of the impact. Invasions may, or may not, have legacy impacts after their removal (Corbin and D’Antonio 2012). Removal of the invader does not necessarily restore all ecosystem properties to pre-invasion conditions (Zavaleta et al. 2001).
3. Invasion impacts are often species and site specific. GIIN allows studying the effect of several invasive plants of various life history strategies and functional traits across diverse systems. This allows for identification of commonalities and idiosyncrasies among invasive species and invaded systems.
4. The cover or abundance of the invader is generally not explicitly considered when estimating ecological impacts, despite examples of known cover–impact relationships (Thiele et al. 2010; Greene and Blossey 2012). With sufficient replication, we can test cover–impact relationships for a broad range of relevant and important ecosystem pools and fluxes across a range of species and systems, offering insight not previously available.
5. The scale of detection (plot size) must match the invader life form (e.g., herbs, shrubs, trees). Thus, our method is flexible and can be adapted to different invaders and ecosystems.

## Field design

The methods outlined below include guidelines on site selection, treatment layout, plot maintenance, and impact quantification scalable to any species and ecosystem.

**Figure 1:**
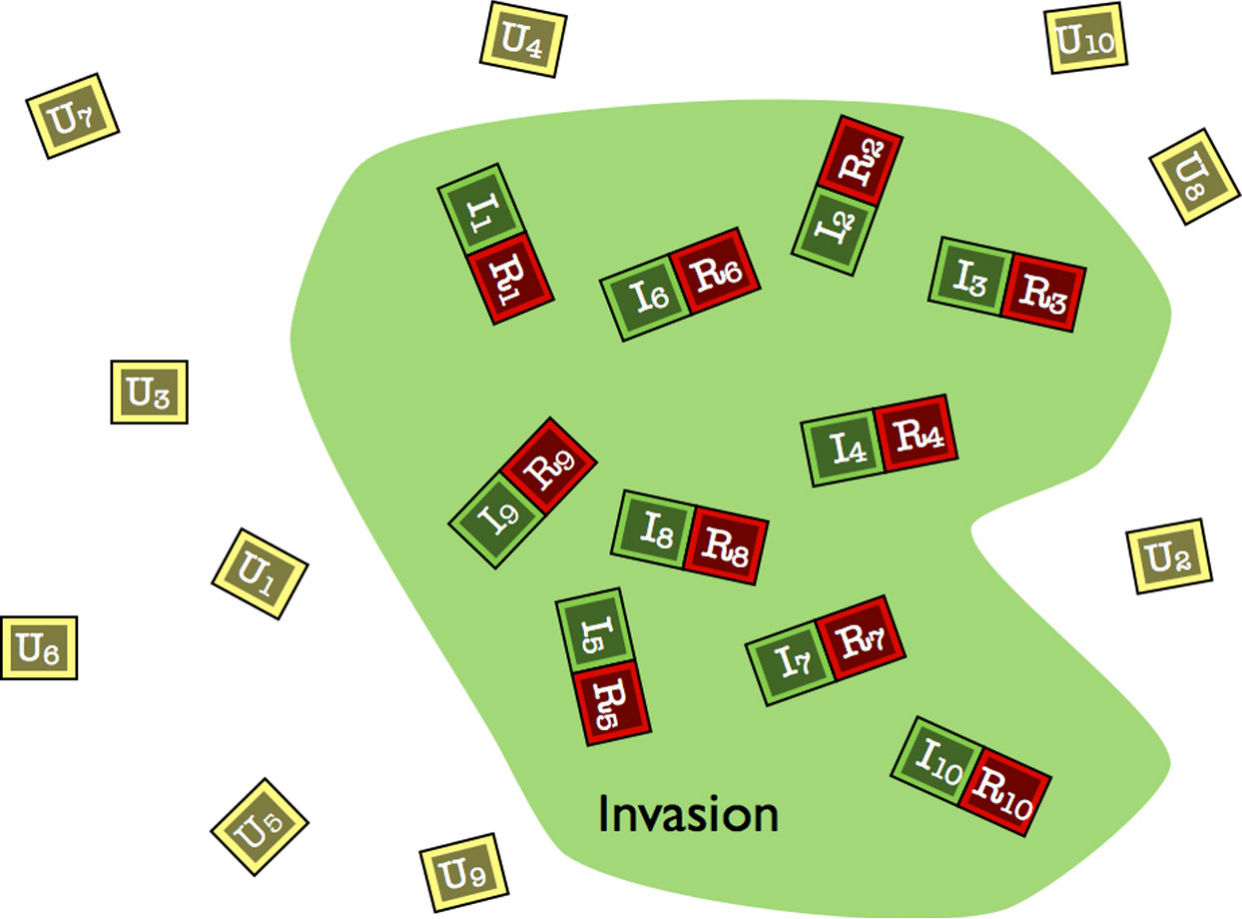
Randomization of invaded (I_n_), invader removal (R_n_), and uninvaded (U_n_) plots in a single invasive population. Invaded and removal plots within pairs should be randomized as well (not shown). The minimum of 10 of each quadrat type is shown.

*Site selection and site-level data collection*:

1. Each site needs to be relatively homogeneous and representative of a particular ecosystem (e.g., deciduous forest, tallgrass prairie), without expected infrequent large-scale disturbances (e.g., fire, flood) unless the disturbance is a determinant of plant community dynamics (e.g., succession that has a particular stage vulnerable to invasion). Identify a site that is invaded predominantly by a single species to reduce interactive (synergistic or antagonistic) effects of multiple species on impacts of interest to the extent possible. Each site should be large enough to accommodate the experimental footprint and include random selection of candidate plots (see below, Fig.1). Many invasion impact studies are conducted in areas with >50% cover of the invasive species (Vilà et al. 2011), although each site should be representative of a “typical” invasion, in terms of the population characteristics of the target invader for that system.
2. Sites should be chosen where enough space exists outside the target invasion to serve as the uninvaded (reference) site that is environmentally similar (i.e., similar slope, aspect, vegetation, land-use history), does not cross any major boundaries (e.g., river, habitat edges), and is not invaded. Finding a suitable reference area adjacent to the invaded area is important to drawing appropriate conclusions on longer term invader impacts. The reference site should be capable of being invaded and not have any obvious reason why the invader is not there beyond dispersal limitations.
3. More than one spatially separate population is strongly encouraged but not required, as the ability to estimate among-population (or intraspecific) variation would be beneficial. Single populations can be included, but the overall aim of the network is best met with data from multiple populations of each study species.
4. Walk the perimeter of the invaded area with a GPS to generate an area (GIS polygon). Population boundaries should be estimated based on your study system. For example, a sterile clonal forb may have a smaller isolation distance (i.e., distance separating two populations) than an outcrossing grass. Include the population size in your dataset, and specify your isolation distance. Reassess the population size every 3 years with attention to average horizontal error.
5. Record the following for each of your populations:
  - state, province, or territory, and country;
  - latitude, longitude (decimal degrees), and elevation;
  - ecosystem (e.g., deciduous forest, old field, grassland, riparian);
  - target invader name (Latin binomial with authority), subspecies, variety or any intraspecific taxonomic unit (when relevant), life form (e.g., biennial, annual grass, deciduous tree), native range;
  - target invader patch size (m^2^). Where available a GIS shapefile of the study population(s);
  - invader’s residence time in the patch, if this information can be obtained.

## Experimental design

### Plot size

The plot size should scale with the size of the target invader and should be based both on invader size and logistical constraints. Plots should be at least 1 × 1 m for most grasses and forbs, and we suggest 5 × 5 m, 10 × 10, or 20 × 20 m for shrubs and trees. Ecological impact metric data are collected in each plot, which will be surrounded by a border to reduce edge effects. Removal plots should have a border proportional to the height and root system of the invader and be at least half the extent of the plot size. For example, the border for common reed (*Phragmites australis*) would likely be much larger than for garlic mustard (*Alliaria petiolata*) due to size differences and life history-related characters of size and underground organs.

## Observational study

The observational component allows comparison of the impact of a single invader on the study system relative to an adjacent uninvaded area (invaded vs. uninvaded).

1. Within each invaded site (isolated population), locate ≥10 randomly, or stratified random if the invader is typically patchy, appropriately sized plots (include appropriate border size) that are within the invasion and are representative of the site (Fig.1).
2. The “Invaded” plots will not be manipulated and serve as the observational component.
3. Mark the corners of the plots with a permanent marker (e.g., nail, rebar, stake, or high-precision GPS) to ensure relocation in subsequent years. Record impact at the same location over time (≥3 year) to identify temporal shifts (or stability) of recorded metrics.
4. Locate an equivalent number of “Uninvaded” plots randomly outside the invaded area ensuring that the plots are located in the same environment (i.e., same aspect, slope, habitat, community type, successional stage, disturbance) as the target invader, yet far enough from the invader to insure it is not invaded throughout the duration of the study or receive impact from the invader (e.g., shade).
5. Collect data from each plot at peak community productivity in each system in a relatively short period to avoid differences among plots due to seasonal vegetation dynamics (see Tables1 and 2 and Impact metric sampling below).

**Table 1:**
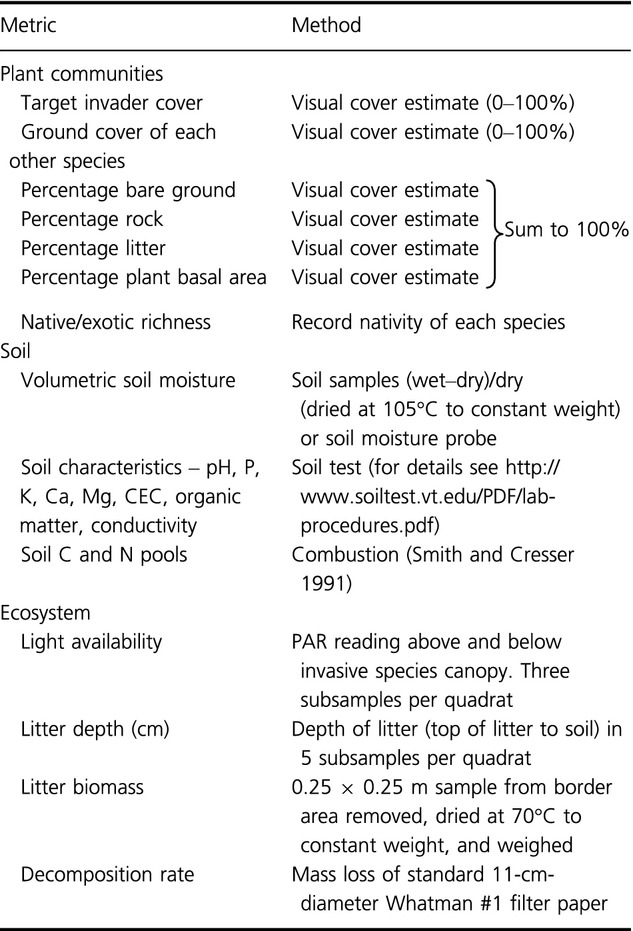
Core measurements to be made in all plots. Several additional metrics will be derived from the measured quantities listed below (e.g., diversity index, H’)

**Table 2:** Optional data collected where possible

Metric	Method
Soil micronutrients (Zn, Mn, Cu, Fe, B)	Soil test (for details see http://www.soiltest.vt.edu/PDF/lab-procedures.pdf)
Available nitrate, ammonium, and phosphate	IER resin bags (5 g Duolite) buried at 5 cm installed in spring (“harvested” after 1 year)
Microbial biomass	Chloroform fumigation
Soil microbial C, N	“direct extraction” of soil cores, 1 per quadrat
Microbial activity	Basal respiration is used as a surrogate of activity. 1 g soil collected from each soil strata (0–5 cm and 5–10 cm) placed in 20-mL serum bottle with 50 *μ*L of water added and acclimated overnight. Then, 2 mg glucose g^−1^ field moist soil added and samples incubated at room temperature for 4 h. 0.5 mL headspace gas extracted and measured in a gas chromatograph per hour for 4 h. Additional infield measurements are also encouraged. For example, Li-8100A (Li-Cor, Lincoln, NE)
Earthworm richness, biomass	2 L of a 9 g L^−1^ yellow mustard solution applied to 10 cm PVC rings driven 5 cm into ground, 2 per quadrat. Count emergent earthworms within 5 min, store, dry, and weigh
Nitrogen mineralization rate	Two identical soil cores for incubation (28-day incubation) and N analysis (ISO 14238, 1997)
Litter nutrient content	Tissue nutrient analysis (5 pooled subsamples per quadrat)
Total litter C, N, P, and C:N, N:P, C:P	Analysis of 10 × 10 cm sample of litter collected following the growing season. C, N – combustion according to Smith and Cresser 1991; P – dry-ashing and extraction using hydrochloric acid with vanadomolybdate procedure (Jackson 1958) and spectrophotometric analysis
Litter-cellulose Index (LCI)	Acid detergent fiber and neutral detergent fiber methods, which utilize proximate C fractionation analyses (Goering and Van Soest 1970). Calculate LCI = lignin/(lignin + cellulose)
Arthropod richness and abundance	Pitfall traps, or litter sieving
Soil compaction	Soil penetrometer, 3 subsamples per quadrat
Soil infiltration rate	Use 10-cm-diameter pipe installed 8 cm into soil. Volume of water used should be adequate to calculate a rate
Select native species fitness	Collect seed output per individual for 5 individuals in each quadrat
Seed bank analysis	Soil samples collected each year with identity and number of seeds. Combination of greenhouse grow-outs and elutriation

## Manipulative study: invader removal

The target invader removal component allows an expanded hypothesis to be tested of the legacy effect of the target invader on the study system, while also providing an additional reference against which to compare the invaded plots. This requires continuous, or at least annual, removal of the target invader to address possible legacy effects.

1. The layout is the same as above with the addition of a paired plot next to the “Invaded” plot, with invaded and removal plot assignment randomized (Fig.1).
2. Within each “Removal” plot, manually clip at ground level only the target invader on an as needed basis to ensure ˜0% ground cover throughout the duration of the study, including the border. Clipping at ground level reduces soil disturbances that can cause unintentional effects and confound impact metric interpretation. For some species, the invader will need to be removed more than once per season to ensure complete removal.
3. Record the same metrics and timings as above.

At this time, the GIIN protocol is limited to removing only the target invader, and leaving other exotic species, including those that may colonize following target invader removal. This allows attribution of any ecological impacts to a specific species. Additional objectives may include removal of all exotic species, which would test other important hypotheses regarding the impact of exotic plants (see below for additional complementary objectives that could be added to GIIN).

## Sampling

Key features of the community and ecosystem were selected to represent important pools and fluxes that are commonly associated with terrestrial invasive plants (Ehrenfeld 2010; Vilà et al. 2011). The included metrics are relatively simple to measure, cost-efficient, and implicated in ecosystem function. All coordinated distributed experiments require a balance of sample depth and breadth with participant expense and expertise (Colautti et al. 2014). Therefore, we include both core (Table1) and optional measurements (Table2; see also Kumschick et al. 2015).

Most data will be collected within the interior of each plot to reduce edge effects. While invader removal treatments are implemented throughout the year, measurements are made and samples collected at peak community productivity, which will vary among systems.

- *Vegetation*: Collect visual estimates of percent ground cover for all vascular plant species within each plot. Include a skyward fisheye photograph or other objective measurement of tree cover when studying tree invasions and monitor accordingly.
  - Record ground cover and height estimates for all species in all vegetation layers. Two observers estimate the percent ground cover to the nearest 1% and take the mean of the estimates for each species, record species nativity, and the structural layer in which it occurs. Also record nonvegetated areas (e.g., bare soil, rocks, etc.) for the ground layer. Measure or estimate the height of each species, which can be used to calculate volume, which is highly correlated with biomass.
- *Soil*: On the edge of each plot, collect five randomly located soil samples to 10 cm deep using a standard 2.25 cm diameter soil corer. Soil should be sampled after at least 48 h without rain. Homogenize and pool samples in plastic bags keep them cold and analyze soon after collection. For determination of macro and micronutrients, soil samples should be analyzed using Mehlich I extractant (Maguire and Heckendorn 2011).
  - Basic soil tests include pH, phosphorus, cation exchange capacity, electrical conductivity, and organic matter. Micronutrients (Zn, Mn, Cu, Fe, B) are presented as optional analyzes. Pass fresh soil through a 2-mm sieve.
  - Total soil carbon and nitrogen content samples should be air-dried subsamples from the pooled samples above and finely milled (0.1 mm).
- *Decomposition*: Place two 11-cm-diameter Whatman #1 filter paper to fit in a 14 × 14 cm mesh bag (1 mm mesh size). Cut polyester mesh into 14 × 28 cm strips, place the preweighed filter paper (dried at 70°C to constant weight) inside, fold and stitch together using polyester thread or stainless steel staples. Place three bags per plot along the edge of the plot below the existing litter layer and secure to the soil with stainless steel nails. Retrieve one bag after 3, 6, and 12 months of incubation. Record the mass at *T*_0_ of the filter paper as the starting mass to determine a mass loss. Place litter bags in plots in late spring, when you would be doing removals. Upon collection oven dry at 70°C to constant weight, remove the filter paper, record the mass, and perform an ash correction (incineration at 550°C for 5 h).
- Additional core metrics should be collected as specified in Table1.
- Table2 lists additional metrics of interest that could be collected as well to expand the depth and breadth of ecosystem impacts.
- Include a link to the closest weather station for access to local data. When resources are available, set up temperature and humidity sensors (e.g., HOBOs, iButton), this is especially important to test for environmental microsite differences.
- Photographs of representative plots should be recorded annually.

## Data analysis

**Figure 2:**
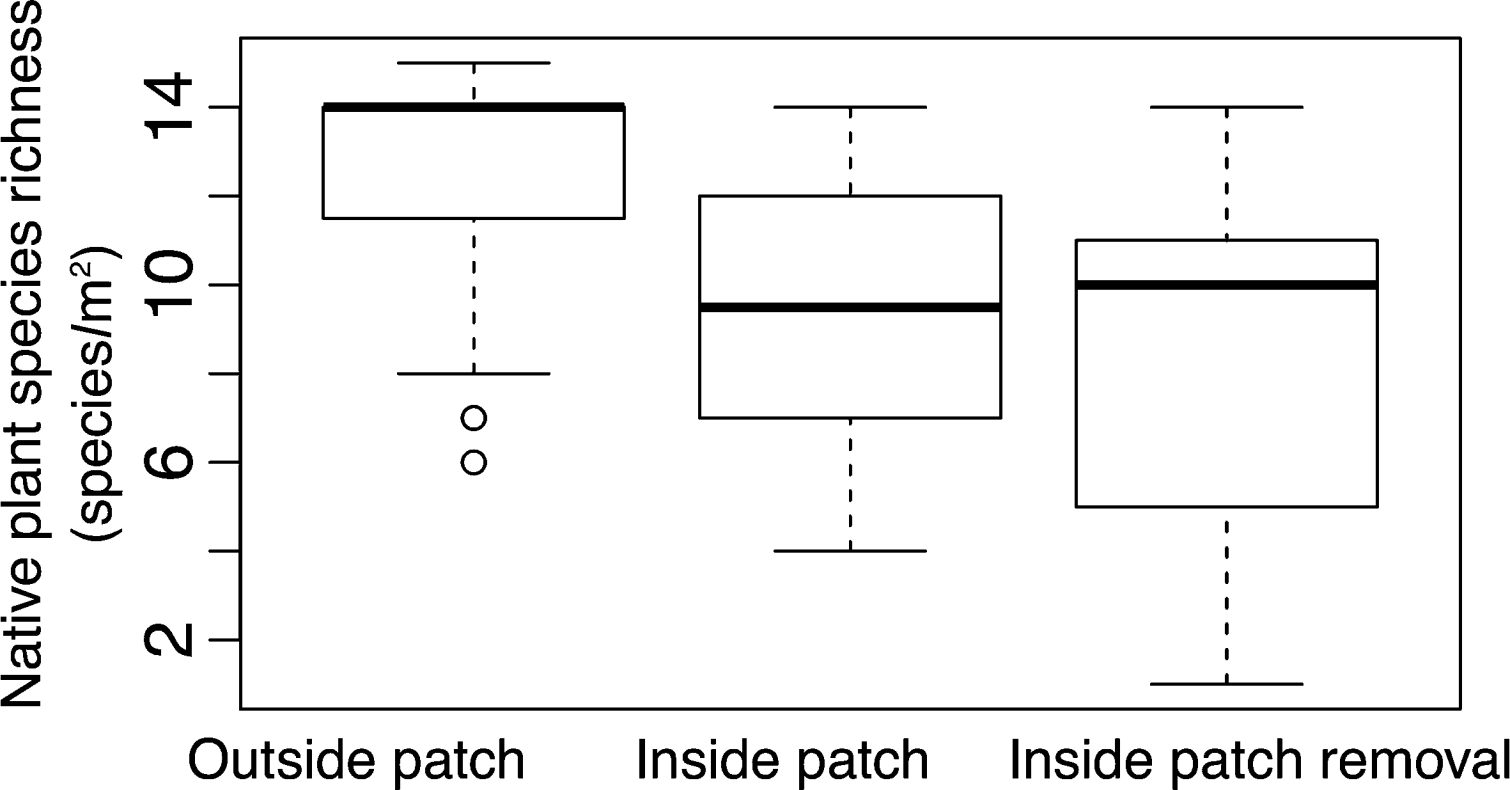
Median native plant species richness and evenness (bold horizontal line) from outside the patch (uninvaded), inside the patch (invaded), and inside the patch with the invader removed (removal). Boxes around medians are 50% of data, whiskers are approximately 2 standard deviations from the median and points (empty circles) are outlier values, which in this case are below the first quartile of the distributions. Data are simulated, and in this case, there was a significant treatment effect comparing means with ANOVA which was due to significant differences between the outside invader patch and inside invader plots. There was no difference in species richness between the inside and outside removed treatments indicating a possible legacy effect, although there was no intentional legacy effect placed into the data creation.

**Figure 3:**
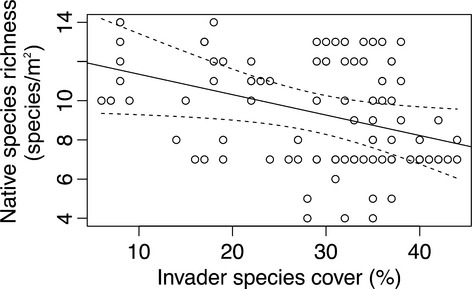
Relationship between invasive species cover and native species richness in plots from outside the invader patch and inside the invader patch, but not including the invader removal treatment. The linear regression was significant (*P* < 0.001, adj. *r*^2^ = 0.282) and predicted line and 2 × SE lines (dashed) shown.

Several comparisons and levels of analyses are possible using the GIIN design. The difference between invaded and uninvaded plots will be evaluated, with differences attributable to the target invader, assuming the uninvaded area is invasible and not inherently different (Fig.2). Removal plots will additionally be compared to invaded and uninvaded plots to identify possible legacy effects (Fig.2). Strong invader legacy effects would be revealed in removal plots failing to converge on uninvaded plots (again the validity in the assumption that uninvaded plots did not differ systematically from invaded plots at the time of the invasion). Each metric could be compared singly among treatments at each site, grouped into functional attributes (e.g., nutrient pools) (Vilà et al. 2011). Multivariate methods could also be employed to integrate across metrics to compare differences among invaded, uninvaded, and removal across time (e.g., Barney et al. 2013). Data can be analyzed within an individual study, or across studies, with various levels of nesting included as appropriate. With sufficient data from different sites, climatic variables could be included to evaluate whether and how patterns of impacts vary across regions from different bioclimatic zones. For species with adequate spatial replication, we could also explore the relationship between invader cover and response variables (Fig.3), which is predicted to be nonlinear (e.g., McCarthy 1997), but is rarely explored empirically (Barney et al. 2013). An important advantage of the GIIN protocol is allowing investigation of invader impacts with respect to invader cover (Fig.3; e.g., Thiele et al. 2010) and environmental co-variation (Thiele et al. 2011). These relationships could be explored using a variety of models and assumptions (e.g., linear, nonlinear; Thiele et al. 2011). Importantly, relative differences between the treatments can be evaluated singly, as has been performed with the overwhelming majority of existing impact studies (except see Thiele et al. 2010), but also over time, which is less common.

## Discussion

### Advantages of standard methods and measurements

A large number of ecological hypotheses, theories, and the effects of environmental drivers are being addressed with meta-analyses, which synthesize results of different studies to test for generalizability. Koricheva and Gurevitch (2014) identified 322 ecological meta-analyses in the last 14 years, including several on invasive species (e.g., Vilà et al. 2011). Despite the many advantages of meta-analyses, they suffer from several disadvantages: differences in experimental design, publication bias, and lack of reporting (e.g., Levine et al. 2004; van Kleunen et al. 2010; Vilà et al. 2011). Meta-analyses often identify knowledge or data gaps that preclude the ability to generalize, while coordinated distributed experiments can “produce insights unavailable using other approaches” (Gurevitch and Mengersen 2010; Koricheva and Gurevitch 2014). Distributed experiments use standard methods and are increasingly employed to address ecological questions and refine causative hypotheses in a robust way across the globe (Borer et al. 2014a). Invasive species are commonly included as a major ecological challenge (Fraser et al. 2012) and certainly are a global issue (Millennium Ecosystem Assessment 2005). Therefore, determining the ecological impacts of invasive species is a compelling issue to study using a coordinated distributed experiment.

Individual studies of impact typically record few metrics as they are often hypothesis-driven and focused on answering specific questions (Hulme et al. 2013). For example, studies on *Berberis thunbergii* have focused on nitrogen cycling (Cassidy et al. 2004), soil microbial community dynamics (Elgersma et al. 2011), and earthworm interactions (Nuzzo et al. 2009) to address specific ecosystem aspects hypothesized to be affected by this exotic shrub. While this is a powerful way to understand a specific feature of an invasion, the consequence of such a focused approach is a weaker understanding of the broader context of *B. thunbergii* in North American eastern deciduous forests. Additionally, the unchanged, or unanticipated changes, to other ecosystem pools and fluxes are as important as understanding those that are changed and contribute to our broader understanding of the role invasive plants play in the ecosystem (Barney et al. 2013). Therefore, GIIN is focused on testing more broadly the ways that invasive plants might impact their recipient ecosystems. However, GIIN is flexible to allow incorporation of hypothesis-driven questions and additional metrics (e.g., adding plant–pollinator network interactions). Ultimately, GIIN will refine hypotheses of plant invader impacts across a range of species and offer a basis for higher certainty in inference space on causative factors that can only be determined with wide geographic distribution of common protocol experiments.

**Figure 4:**
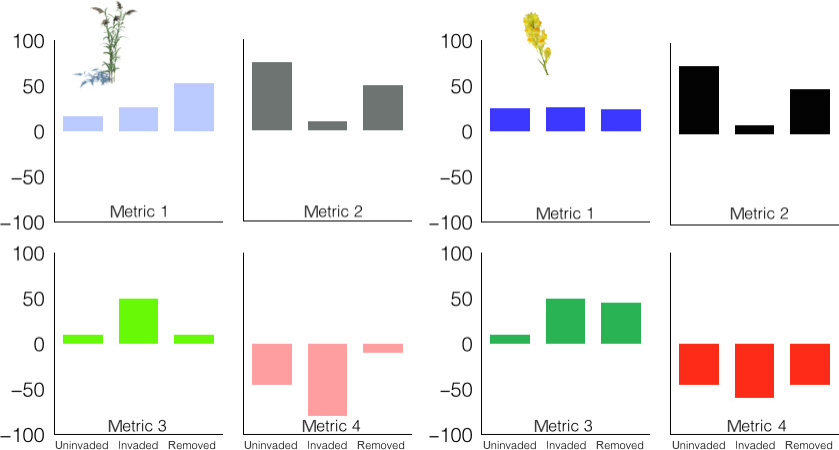
Comparison of four hypothetical responses between two populations of two species in uninvaded, invaded, and removed plots among four hypothetical impact metrics (1–4). The standard methods allow direct comparisons regarding impact variability (metric 1), directionality (metric 2), legacy (metric 3), and magnitude (metric 4).

Standard metrics allow direct comparisons of the magnitude, direction, and legacy of individual effects among species (Fig.4 row 1 and row 2) and among populations within a species (Fig.4 column 1 and column 2). Population-level variation in ecological impacts is rarely studied (Barney et al. 2013), as most studies occur at a single location (e.g., Alvarez and Cushman 2002). Even the Parker et al. (1999) method does not account for within species spatial variation. Thus, we know little of how spatially stable or variable impact is among populations. Population-level variation is of interest itself, but also allows for estimations of species-level impact by examining the variation in impacts across sites (populations). For example, to date, there are four populations of *Microstegium vimineum* being studied using GIIN ranging from southwestern Virginia to Connecticut.

Sites may vary in their “starting point” (uninvaded values), which can affect the magnitude of change by the invader. Therefore, percentage changes could be calculated for each metric in each population, which has also been suggested as an effective mechanism to compare and combine metrics with variable units (Barney et al. 2013). Thus, the magnitude of change can be compared both absolutely and relatively among metrics, populations, and species. Directionality (positive or negative change) will also be of interest, which may affect the interpretation of and uncertainty about the impact on ecosystem services and help direct or prioritize management decisions. For example, in some instances, sediment accretion may be viewed positively (e.g., eroding dunes) or negatively (e.g., tidal estuaries). Broad accounting of standard metrics across diverse systems would facilitate “vote-counting” of directional changes of a particular metric (Pyšek et al. 2012).

Invasive plants are managed to mitigate their ecological impacts in the hopes that the system will return to the pre-invaded state. However, there is evidence that once invasive plants are removed, their ecological impacts or the impacts from the removal method (Ortega and Pearson 2005; Skurski et al. 2013) may persist – termed legacy effects. Corbin and D’Antonio (2012) describe biotic, soil chemical, and soil physical legacies, which may vary in “recovery time” or “return rates.” Some of these legacies may be so strong that the system may never return to the pre-invaded state, but instead achieves a new “post-invasion” state, dominated sometimes by a different invasive species (Skurski et al. 2013; Kuebbing & Nuñez 2014). Therefore, understanding the postinvasion process is as important as understanding the impacts of the extant invasion. The GIIN protocol includes an experimental invader removal treatment allowing quantification of legacies on individual metrics, including all of those outlined by Corbin and D’Antonio (2012).

In their seminal paper on invasive plant impact, Parker et al. (1999) suggested quantifying the per capita impact and scaling to cover the range size of the species. Others have recently suggested that impacts may scale with the density or level of cover of the invader (Thiele et al. 2010; Barney et al. 2013). For example, Greene and Blossey (2012) showed that native species richness and performance declined linearly with *Ligustrum sinense* cover. However, few empirical studies include or account for invader cover, which may have functional relationships that may vary by metric or species (Fig.3). With sufficient replication impact relationships can be evaluated with invader cover (Catford et al. 2012; Hejda 2013), which may facilitate identifying management thresholds (e.g., populations should be managed while cover is <25%).

**Figure 5:**
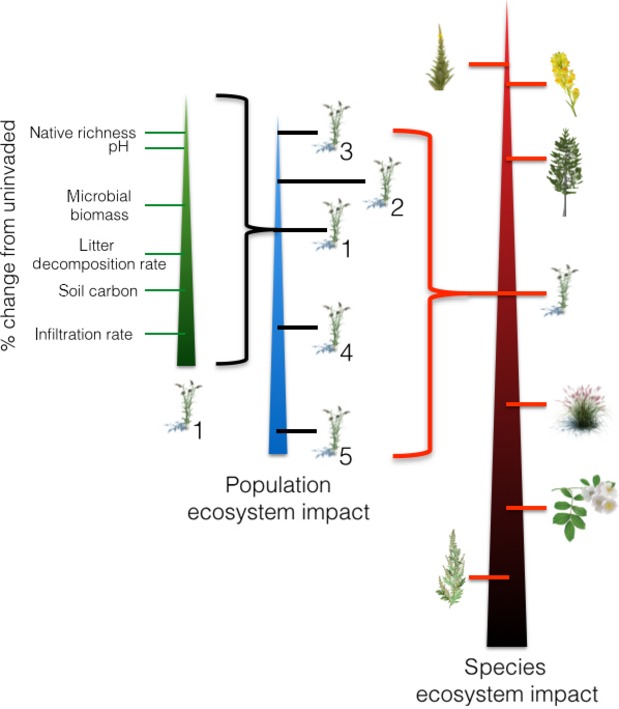
The GIIN methodology allows ranking of impact at several scales and levels of organization. Here we show the relative impact (as a percent difference from the uninvaded) among metrics within a population (left scale), the relative integrated population ecosystem impact of five populations (middle scale), and relative rank of mean species ecosystem impact among several invasive plants. The species depicted here do not reflect actual impacts.

Despite rankings of the “world’s worst invaders” or “top 100,” there is no empirical mechanism to know which invasive plants have more impact than others (Lowe et al. 2004). The GIIN protocol allows for relative comparisons to be made within a population, among populations within a species, and among species (Fig.5). For each population, the percentage difference from the uninvaded plots can be calculated to allow relative comparisons among metrics with different units to identify which components of the ecosystem are being most impacted. Populations could be ranked and compared using an integrated impact metric such as that proposed by Barney et al. (2013), which integrates any number of impact metrics into a single population-level value (but see Hulme et al. 2014). If several populations were sampled, the variation in “total” impact could be assessed. The same could be performed with individual metrics if a more targeted hypothesis-driven approach is desired (Hulme et al. 2013). Lastly, the mean species–impact (average of all sampled populations) could be compared among species to identify which species are most impactful within a given set of standard metrics (Fig.5).

This proposed framework should be viewed as a starting point as additional hypotheses, and relationships could be explored and build on this design. For example, adding additional plots in which all exotic species are removed to explore the broader context of invasive species and how they interact (Kuebbing and Nuñez 2014). The relationship of invader density to impact could be further explored by adding additional plots along a density gradient from low to high cover. Additional plots could be added to the invasion edge where density is often higher, particularly for herbaceous clonal species (Lehnhoff et al. 2008). The GIIN protocol is nondestructive, but the effects on net or ecosystem primary productivity could be explored with additional plots that are harvested. This design allows for uniformity to test basic hypotheses, but flexibility to test emerging hypotheses of interest.

### Disadvantages of standard method/metrics

Coordinated experiments are not without their limitations, and GIIN is no exception. Unlike other coordinated distributed experiments, our methodology requires different plot sizes depending on the target invader. Comparisons among species would assume that impact would scale appropriately with plot size. For example, the plot size for the small understory annual grass *Microstegium vimineum* is 1 × 1 m, while that for *Pinus contorta* is much greater. The larger plot size is necessary to accommodate species of various sizes and life histories, although there is no general rule for selecting the appropriate scale for impact studies. However, most previous experimental impact studies are conducted at comparable scales to what we propose here. There is clearly an important logistic and funding limitation to larger plot size and higher replication. Thus, each study should consider the design that can be monitored in the long term. An extremely expensive and complex setup may not be realistic when considering the long-term costs of monitoring, especially if equipment (e.g., weather sensors) and analyses (e.g., soil) are considered.

Our approach covers many important community, soil, and ecosystem metrics, but is not comprehensive (see Kumschick et al. 2015). As Borer et al. (2014a) suggest, network protocols must be simple and inexpensive to execute to ensure broad participation and reliable data. Thus, many important metrics are not accounted for in the obligatory metrics, but are covered under the optional metrics that participants are encouraged to monitor (Table2). There is always a trade-off between generalization and depth, GIIN can help obtain general and global information, but may fail to provide evidence to answer more specific questions. Additionally, some researchers may be interested in more focused hypothesis-driven ecological impacts (Hulme et al. 2013). This would not be mutually exclusive from the GIIN methodology, but could be performed in addition to GIIN. There is also potential bias in data collection caused by different teams of researchers conducting the same protocol in different places. The populations included in GIIN may vary in their stage of invasion; some may be quite new, while others may be very old. In many cases, the age of the invasion is unknown, except for many woody species and more rarely in forbs (see Dostál et al. 2013 for an example). This variation in starting points may affect the magnitude of measured metrics. However, since GIIN plots are followed through time, we may be able to identify invasions that continue to accrue impacts – perhaps suggesting a young invasion – and others with more stable metrics. Consistent trends in metrics are more important to consider than absolute values across studies.

## Conclusions

To understand the causes and consequences of species invasion, it is imperative to understand the impacts of these species on ecosystem pools and fluxes. Such an understanding will better inform management and policy. Addressing this and other ecological grand challenges requires collaboration and standardized protocols in search of generality. The low-cost GIIN protocol presented here is designed to give invasion scientists insight into both local, population-level impacts, but also make broader relative comparisons among diverse species invading a variety of global ecosystems. The GIIN protocol is flexible enough to allow for necessary adaptation to site characteristics and species type, but maintains the capacity to answer the same key questions in different systems avoiding the problems of standard meta-analyses. This protocol also offers the flexibility of making comparisons between the invaded site to adjacent uninvaded sites, but also to invader removal sites, facilitating quantification of current and legacy impacts. Invasive plant managers lack the tools for quantifying impacts so that they can prioritize species and populations of species for management. Land managers and policymakers would benefit from increased quantification to establish management trigger points. A more complete and standardized knowledge of the breadth and depth of impacts to design effective, efficient, and durable invasive plant management strategies are a clear need to mitigate one of the drivers of global change.
